# An unusual huge thymoma composed of sclerosing thymoma and type AB thymoma: A case report

**DOI:** 10.1097/MD.0000000000027873

**Published:** 2021-11-19

**Authors:** Yu-ting Jiang, Tian-yue Zhang, Dan-dan Guo, Rui Li

**Affiliations:** a Sichuan Key Laboratory of Medical Imaging, and Affiliated Hospital of North Sichuan Medical College, Nanchong, China; b Department of Radiology, Deyang Peoples’ Hospital, Deyang, China; c Nanchong Hospital of Traditional Chinese Medicine, Nanchong, China.

**Keywords:** mediastinum, sclerosing thymoma, thymoma

## Abstract

**Rationale:**

Sclerosing thymoma (ST) is quite a rare disease, as denoted in previous literature. Less than 20 cases of ST have been reported to date. However, the combined thymoma, composed of both type AB thymoma and ST, has never been described before.

**Patient concerns:**

The subject, a 49-year-old woman, came in with the chief complaint of cough for 10 days.

**Diagnoses:**

Both the contrast-enhanced computed tomography scan and the ultrasonography showed a huge mass located in the right thoracic cavity with inhomogeneous contrast accompanied by the invasion of the pericardium and pleura. Subsequently, computed tomography-guided core-needle biopsy revealed type B2 thymoma, and type AB thymoma could not be excluded. Based on postsurgical histopathology and immunohistochemical finding, this tumor was given the final diagnosis of ST and type AB thymoma.

**Interventions:**

After 6 months of adjuvant chemotherapy and local radiotherapy, total thymectomy was performed.

**Outcomes:**

The patient has been duly followed up for 1 year without any tumor recurrence.

**Lessons:**

ST is a very rare mediastinal neoplasm. Moreover, ST in combination with AB thymoma and affecting a large area, is unprecedented. Whether radiotherapy and chemotherapy have a certain effect on ST requires further investigation. In addition, due to the unclear recurrence rate of ST, long-term follow-up evaluation seems necessary.

## 1 Introduction

The incidence of thymoma is about 1.5 cases per million and it is the most commonly occurring tumor in the anterior mediastinum of the chest. Sclerosing thymoma (ST) is a rare anterior mediastinal neoplasm, originating within the epithelial cells of the thymus and accounts for an estimated 0.02% of all thymoma.^[1,2]^ The World Health Organization classification of thymomas, divided rare thymoma into micronodular, microscopic, and ST.^[3]^

ST is an extremely rare thymoma with exuberant collagen-rich stroma. It was reported first in 1994 by Kuo,^[4]^ and <20 cases have been reported since then. The clinical manifestation for ST is myasthenia gravis, but approximately 75% of ST patients have no obvious symptoms.^[1]^ The imaging of ST exhibits features of conventional thymoma lacking any special signs; therefore, the final diagnosis mainly depends on the pathology and immunohistochemistry results.

The following is a case of a 49-year-old female patient with ST with a large mass in the right thoracic cavity. This case presentation of the rare neoplasm is for educational purposes, for discussing the relevant pathologic and imaging features, and establishing the diagnosis for future clinical practice.

## 2 Case presentation

A patient is a 49-year-old woman who was admitted with a chief complaint of cough for 10 days without myasthenia gravis, vomiting, nausea, or any other symptoms. The patient was admitted for cough for 10 days. No other conditions were recorded at the time of admission. No significant history of past illnesses was identified. The patient had no previous or family history of a similar illness. During the physical examination, she was found to have a distended abdomen with no palpable masses. Laboratory tests revealed mild leukocytosis (10.4 × 10^9^ cells/L), with normal hematocrit and platelet count. Conventional tumor markers, including CEA, CA199, and NSE, were reported in the normal range.

**Figure 1:**
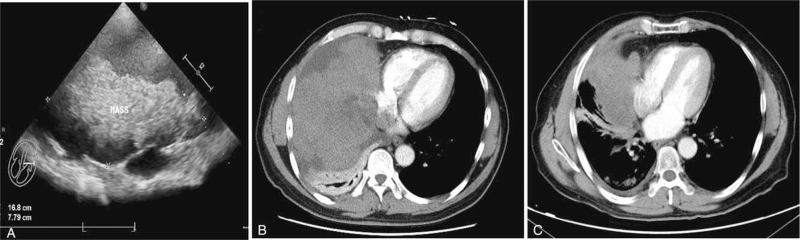
(A) Ultrasonic images: a huge inhomogeneous mass in the right thoracic cavity accompanied invasion of the pericardium. (B) Computed tomography images. A heterogeneous, macro-lobulated mass with inhomogeneous contrast accompanied by partial calcification in the right thorax and mediastinum; (C) After radiotherapy and chemotherapy regimen, the size of the tumor decreased significantly.

Ultrasound examination illustrated a large heterogeneous lobulated mass in the right thoracic cavity, with dimensions 16.8 cm × 7.8 cm, accompanied by invasion of the parenchyma and expansion of the right atrium (Fig. 1A). CT showed a heterogeneous, macro-lobulated mass accompanied by partial calcification in the right thorax and mediastinum, with enhancement (Fig. 1B). No other abnormality was seen in the liver, pancreas, and other visceral organs. CT-guided core-needle biopsy revealed type B2 thymoma, which did not exclude the type AB thymoma. Thus, it was diagnosed clinically as T3N2M0 stage IV B thymoma. Initial treatment comprising of adjuvant chemotherapy and local radiotherapy was performed before surgery, to shrink the size of the thymus tumor. The tolerance to certain doses of radiotherapy in thymoma at risk determines the safety of radiotherapy. A conventional course of intensity-modulated radiotherapy consisting of four sessions a week and a total dose of 70 Gy was administered to the patient, modulated according to the size of the lesions. After radiotherapy, the patient was treated with six cycles of CAP (cyclophosphamide 500 mg/m^2^, doxorubicin 50 mg/m^2^, and cisplatin 50 mg/m^2^ q3w) chemotherapy regimen. Six months later, tumor size significantly decreased with dimensions 3.6 × 2.5 cm (Fig. 1C). Subsequently, total-thymectomy was performed and the entire mass was successfully excised.

**Figure 2:**
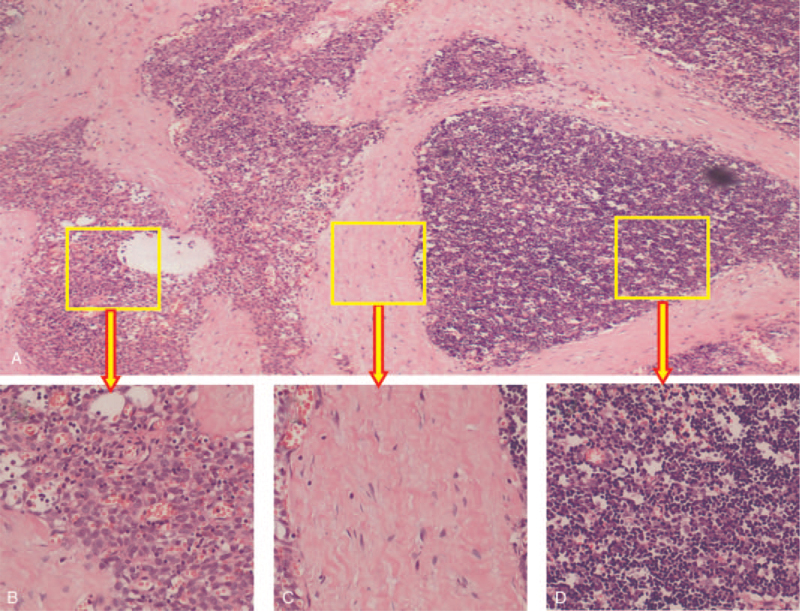
Pathological images. (A) Microscopically, the 3 types of tumor cells are closely arranged and cytologically atypical (hematoxylin-eosin staining, × 100). (B) The type A region of the thymoma consisted mainly of oval tumor cells arranged in nests with a few dispersed lymphocytes (hematoxylin-eosin staining, × 400). (C) Proliferative fibrocollagen is embedded in the aggregation area with some fibroblast cells (hematoxylin-eosin staining, ×400). (D) The type B area consisted mainly of lymphocytes with a few small polygonal epithelial cells with bland nuclei (hematoxylin-eosin staining, ×400).

**Figure 3:**
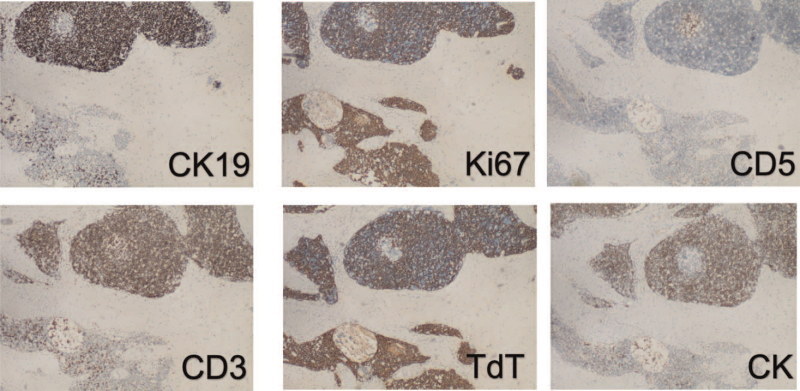
The tumor shows positive CD10 immunoreactivity. Immunohistochemical images: CK and CK19 were positive in the cytoplasm of the epithelial cells in both the type A and B areas of type AB thymoma (×40). The Ki67 index in squamous cell carcinoma was approximately 80% (×40). The immunostaining patterns of CD3, CD5, and TdT indicated immature T-lymphocyte cells in these areas (×40).

The grayish-white tumor was solid-cystic and covered by an incomplete membrane. The solid area with fish-flesh to tan-brown appearance was accompanied by calcification, whereas the cystic lesion appeared as multiloculated, with a slightly rough inner wall and loss of contents. Microscopically, the histologic analysis of hematoxylin and eosin staining revealed that the tumor which was composed of the type A region of the thymoma consisted of a few dispersed lymphocytes, the type B area consisted mainly of lymphocytes with a few small polygonal epithelial cells and extensive sclerotic lesions with hyalinization and calcification (Fig. 2). Pathologically, this tumor consists of 40% sclerosing thymoma and 60% AB thymoma. The margin of the tumor consists of types A and B. For further confirmation, the sections were incubated overnight at 4°C with the primary antibodies CK and CK19, which were positive in the cytoplasm of the epithelial cells in both the type A and B areas of type AB thymoma (Fig. 3). The Ki67 index in squamous cell carcinoma was approximately 80%. The immunostaining patterns of CD3, CD5, and TdT indicated immature T-lymphocyte cells in these areas. CD20 and CD21 were negative. Based on the abovementioned histopathologic and immunohistochemical findings, the final diagnosis was ST and type AB thymoma.

The patient was discharged from the hospital eight days after surgery. During the most recent 1-year follow-up, the patient remained in remission with no sign of relapse. Follow-up with enhanced CT showed complete removal of the lesion with no sign of recurrence.

## 3 Discussion

Thymoma is one of the most common mediastinal tumors, accounting for approximately 50% of all lesions in the anterior mediastinum.^[5]^ According to the World Health Organization classification of thymic tumors, thymoma could be divided into A, AB, B, and C types, and the rare thymomas into micronodular, microscopic, and ST.^[3]^ Most types of thymomas can be detected in an early stage, due to their specific symptoms including myasthenia gravis, pure red cell aplasia, and hypogammaglobulinemia; however, early diagnosis of ST is relatively difficult because it lacks typical clinical manifestations.

In previous cases, most of the ST tumors were reportedly <10 cm in length and diameter and could be combined with all subtypes of thymoma (Table 1).^[1,2,4–8]^ According to Li et al,^[8]^ only slight signs of type AB thymoma were observed at the peripheral region of ST. However, in this case, the mass volume (16.8 × 7.8 cm) was significantly larger than any previously reported tumors, and a considerable region of type AB thymoma could be found in the tumor. Thus far, no such case has been reported in the literature.

**Table 1:** Summarization of case reports of sclerosing thymoma.

Case	Sex	Age, y	Size, cm	Clinical symptom	Myasthenia gravis	Histopathology findings	Treatment	Outcome	Reference
1	F	39	3.0	Palpitation, Dyspnea	Yes	Type B3, epithelial type thymoma	Surgery	Alive and well, 4 y	^[4]^
2	F	23	2.5	Muscle weakness, difficulty in talking	Yes	Type B1, lymphocytic type thymoma	Surgery	Alive and well, 2 y	
3	F	34	5.0	None	No	7 type B2: cellular aggregates composed of a dual cell population of epithelial cells and lymphocytes, no cellular atypia or mitotic activity. 3 type A: cellular aggregates were characterized by spindle cells with scant eosinophilic cytoplasm and absence of cellular atypia and mitotic activity.	Surgery	Alive and well, 1 y	^[6]^
4	F	62	8.0	None	No		Surgery	Alive and well, 6 y	
5	F	37	6.0	Shortness of breath, chest pain	No		Surgery	Died, pulmonary edema	
6	F	27	5.0	None	Yes		Surgery	Died, unknown	
7	M	58	6.0	None	No		Surgery	Died, congestive heart failure	
8	M	44	5.0	None	No		Surgery	Lost to follow-up	
9	M	56	10.0	None	No		Surgery	Lost to follow-up	
10	M	69	7.0	Shortness of breath, chest pain	No		Surgery	Died, renal dysfunction	
11	M	59	6.0	Shortness of breath, chest pain	No		Surgery	Died, congestive heart failure	
12	M	73	10.0	Shortness of breath, chest pain	No		Surgery	Died, unknown	
13	M	60	2.0	Muscle weakness, difficulty in talking	Yes	Medullary type, slight lymphocytes’ infiltration, nomitotic activity,	Surgery	Lost to follow-up	^[1]^
14	M	77	5.7	None	No	Type AB, epithelial type thymoma	Surgery	Lost to follow-up	
15	M	62	3.1	None	No	Type A, IHC: spindle cells; AE1/AE3+, CD34−, lymphocytes; TdT+	Surgery	Lost to follow-up	^[2]^
16	M	10	7.0	Chest pain	No	N/A	Surgery	Lost to follow-up	^[7]^
17	M	65	4.9	None	No	Type B3?	Surgery	Lost to follow-up	^[8]^
18	M	47	2.0	None	No	scattered, small aggregation of spindle to oval cells, mild lymphocytes infiltrate, no mitotic activity, IHC: AE1/AE3+	Surgery	Lost to follow-up	^[9]^
19	F	49	16.8 × 7.8	Cough	No	Type AB, Ki67 80%, CD3+, CD5+, TdT+, CD20− and CD21−	Chemoradiotherapy, surgery	Alive and well, 1 y	Our case

The pathogenesis and biological behavior of ST remain unclear. Histologically, type AB thymoma is mainly composed of diffuse lymphoid cells and clustered spindle cells (CK-positive), whereas the sclerosing type is mainly composed of abundant collagen fibers and a very small number of lymphocytes.^[1,8]^ The tumors that existed for a long time and involved stimulating fibrogenic proliferation and spontaneous regression caused by tumor degenerative change possibly involves the formation of ST. Moran et al suggested that extensive fibrosis may reflect a change that could be considered as an unusual, extensive fibrosing variant of type A thymoma.^[2,10]^ Furthermore, Ito et al suggested that thymoma with hemorrhage and necrosis could transform into a sclerotic lesion as the necrosis component was absorbed over time to cause fibrosis.^[11]^ As illustrated in the present case, the patient underwent >30 times the sessions of preoperative chemotherapy and radiotherapy performed usually, which may cause partial necrosis or hemorrhage, and secondary extensive sclerotic lesions with hyalinization and calcification. However, ST induced by chemotherapy and radiotherapy has not been reported so far. It is still unknown whether chemotherapy and radiotherapy could have that effect or not. These abovementioned mechanisms have not been completely elucidated yet, and more cases need to be included for further analysis in the future.

At present, the optimal treatment of the most sclerosing thymomas is complete surgical removal. The reported patients had a good prognosis after the resection of the mass. No evidence of recurrence or metastasis was reported.

## 4 Conclusions

In conclusion, we have reported a case of thymoma composed of sclerosing component and a large region of the AB types, wherein the patient received a series of chemotherapy and radiotherapy before surgery. This is the first documented case of ST with an extensive type AB component. However, whether chemoradiotherapy could induce the formation of ST still needs further investigation.

## Author contributions

**Conceptualization:** Rui Li.

**Data curation:** Yu-ting Jiang.

**Writing – original draft:** Yu-ting Jiang, Tian-yue Zhang.

**Writing – review & editing:** Dan-dan Guo, Rui Li

**Conceptualization:** Yu-ting Jiang.

**Data curation:** Yu-ting Jiang.

**Formal analysis:** Yu-ting Jiang, Rui Li.

**Funding acquisition:** Rui Li.

**Investigation:** Dan-dan Guo, Rui Li.

**Software:** Dan-dan Guo.

**Supervision:** Rui Li.

**Validation:** Rui Li.

**Writing – original draft:** Yu-ting Jiang, Tian-yue Zhan.

**Writing – review & editing:** Rui Li.
